# Role of Regulatory Non-Coding RNAs in Aggressive Thyroid Cancer: Prospective Applications of Neural Network Analysis

**DOI:** 10.3390/molecules26103022

**Published:** 2021-05-19

**Authors:** Asumi Iesato, Carmelo Nucera

**Affiliations:** 1 Laboratory of Human Thyroid Cancers Preclinical and Translational Research, Division of Experimental Pathology, Cancer Research Institute (CRI), Cancer Center, Department of Pathology, Beth Israel Deaconess Medical Center, Harvard Medical School, Boston, MA 02215, USA; aiesato@bidmc.harvard.edu; 2 Department of Pathology, Center for Vascular Biology Research (CVBR), Beth Israel Deaconess Medical Center, Harvard Medical School, Boston, MA 02215, USA; 3 Broad Institute of MIT and Harvard, Cambridge, MA 02142, USA

**Keywords:** thyroid cancer, non-coding RNA, neural network analysis, metastasis, anaplastic thyroid cancer

## Abstract

Thyroid cancer (TC) is the most common endocrine malignancy. Most TCs have a favorable prognosis, whereas anaplastic thyroid carcinoma (ATC) is a lethal form of cancer. Different genetic and epigenetic alterations have been identified in aggressive forms of TC such as ATC. Non-coding RNAs (ncRNAs) represent functional regulatory molecules that control chromatin reprogramming, including transcriptional and post-transcriptional mechanisms. Intriguingly, they also play an important role as coordinators of complex gene regulatory networks (GRNs) in cancer. GRN analysis can model molecular regulation in different species. Neural networks are robust computing systems for learning and modeling the dynamics or dependencies between genes, and are used for the reconstruction of large data sets. Canonical network motifs are coordinated by ncRNAs through gene production from each transcript as well as through the generation of a single transcript that gives rise to multiple functional products by post-transcriptional modifications. In non-canonical network motifs, ncRNAs interact through binding to proteins and/or protein complexes and regulate their functions. This article overviews the potential role of ncRNAs GRNs in TC. It also suggests prospective applications of deep neural network analysis to predict ncRNA molecular language for early detection and to determine the prognosis of TC. Validation of these analyses may help in the design of more effective and precise targeted therapies against aggressive TC.

## 1. The Biology of Non-Coding RNAs May Provide a Paradigm Shift in Thyroid Cancer Research

Thyroid cancer (TC) is the most common malignancy of the endocrine system [1,2]. Anaplastic thyroid carcinoma (ATC) is one of the most aggressive and lethal types of cancer [1,3], with a median survival of less than 12 months despite the use of conventional treatments such as surgery, radiotherapy, and chemotherapy [4,5,6,7]. In recent decades, studies have identified genetic and epigenetic alterations in TC that may impact tumorigenesis and progression [8]. Most mutations which occur in TC affect the MAPK or PI3K–AKT pathways, for example point mutations in *BRAF* (*BRAF^V600E^*) or *RAS*, which are fundamental for TC initiation and progression [8]. Some studies using a next-generation sequencing approach showed that ATCs are characterized by the accumulation of several different oncogenic alterations [3,9].

Importantly, the Encyclopedia of DNA Elements (ENCODE) project revealed that non-coding portions of the genome are copied into thousands of RNA molecules [10], and non-coding RNAs (ncRNAs) account for more than 90% of the RNAs from the human genome [11]. ncRNAs can be categorized into classes by size.

Important ncRNAs in cancer include microRNAs (miRNAs), transfer RNA-derived small RNA (tsRNA), and PIWI-interacting RNAs (piRNAs). By contrast, long ncRNAs (lncRNAs), which are characterized as untranslated RNAs greater than 200 nucleotides in length, include subclasses such as pseudogenes and circRNAs [11,12]. Recent studies have discovered that ncRNAs represent functional regulatory molecules that mediate not only cellular biological processes including chromatin reprogramming, transcription, post-transcriptional modifications, and signal transduction, but also play an important role in cancer [11,13]. Furthermore, ncRNAs coordinate complex networks in cancer [13]. Thus, recent findings indicate a paradigm shift in our understanding of cancer biology with respect to deregulated ncRNAs, which are implicated in regulating tumorigenesis and tumor progression [11,12,14].

**Figure 1:**
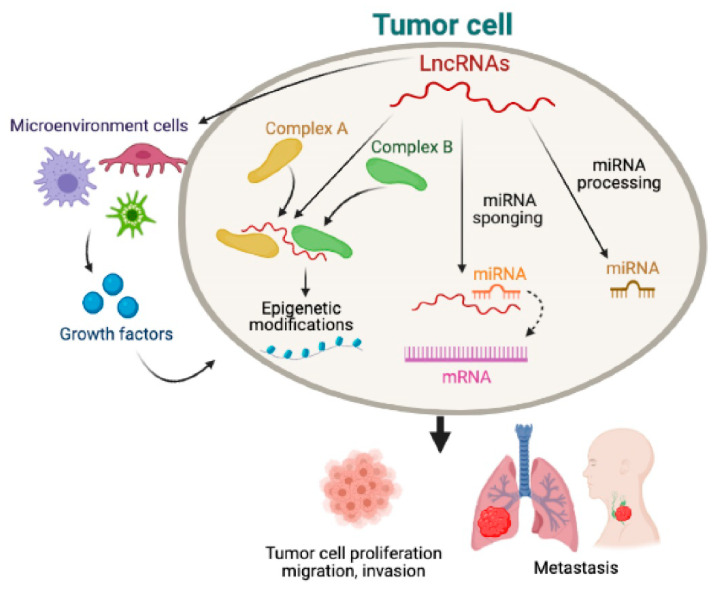
Representative regulatory networks of long non-coding RNAs (lncRNAs) in human tumor cells. Some tumor-derived lncRNAs may be processed into micro RNAs (miRNAs) which can function as tumor suppressors or promoters. LncRNAs may also act as a sponge for some miRNAs which cannot bind to mRNAs. LncRNA may cause genome-wide epigenetic modifications through binding to proteins or protein complexes and control different cellular functions. Tumor-derived lncRNAs also regulate growth factor secretion from the microenvironment of non-tumor cells, leading to metastasis and ultimately to tumor progression.

In this article (Figure 1), we will provide a brief overview of the role of gene regulatory networks (GRNs) of some ncRNAs in preclinical models of aggressive TC. We highlight some examples of lncRNAs coordinating networks of gene interactions. This article overviews the potential role of ncRNA GRNs in models of differentiated TC and ATC and suggests prospective applications for deep neural network analysis to predict the molecular language of ncRNAs for early detection and to determine the prognosis of ATC. Validation of these analyses may help in the design of more effective targeted therapies against ATC.

## 2. Prospective Applications of Neural Network Analysis in Aggressive TC

To understand genetic interactions and assess gene networks in ATC, deep neural network methods for the reconstruction of GRNs may be an effective tool [14]. Reconstructing of GRNs from high-throughput data was considered a challenge [15]. The use of neural network analysis to unravel GRNs is critical for elucidating gene function, depicting biological processes, and designing candidate genes for biomarkers in diseases [16]. Neural networks are a soft computing tool that can be used to learn the pattern from the raw input data similarly to the function of neurons [14]. This model is biologically plausible and noise-resistant [14]. In the neural network analysis, genes are simplified by nodes input or output layer of the neural network [14]. For the neural network method, a more significant and improved formula is used for modeling GRNs by using the Perceptron Learning Rule [14]. GRN can be simplified as nodes which can be coding genes, non-coding genes, proteins, other gene products, etc. Therefore it may form a graphical depiction designed by assessing the behavior of genes and their effects on other genes [14]. Network analysis can also be used to investigate the biology of miRNAs [17]. Therefore, if we can apply neural network-based GRN reconstruction analysis, integrating both genetic and epigenetic data, we might have a powerful method to analyze big data and ultimately elucidate the mechanisms of ATC development and progression.

## 3. Network Motifs

**Figure 2:**
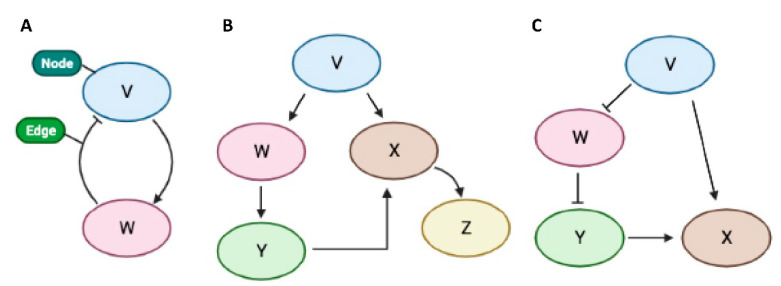
Schemes of a representative gene regulatory network. (**A**) Examples of nodes (coding genes, non-coding genes, non-coding RNAs, mRNAs, or proteins). Arrows indicate activation, whereas T-ending lines indicate repression, creating a feedback loop. (**B**) Representation of a positive feedforward loop. (**C**) Representative feedforward loop including repressive regulation between V, W, and Y.

In a complex organism, cell functions and behavior are controlled by complex networks which regulate gene expression [13,18] (Figure 2). Different classes of networks may be defined by motifs [19]. The canonical network motifs of complex molecular interactions can be represented by regulatory networks composed of nodes and edges [13] (Figure 2). ncRNAs as nodes might link correlative genes into regulatory networks in tumor cells [13]. Interactions between nodes are represented as edges [13]. Furthermore, nodes with a significant number of connections, e.g. miRNAs and transcription factors, represent network hubs [13]. Because ncRNAs such as lincRNA (long intergenic ncRNA) and miRNA have diverse targets and their interactions vary depending on the cell type [13], it is more effective to approach functions of ncRNA by the construction of network motifs.

## 4. Roles of ncRNAs in Canonical Network Motifs

It is challenging to accurately predict the function of ncRNAs as tumor promotors or tumor suppressors [20]. The diverse ability of ncRNAs can be presented through the distinct gene production from each transcript as well as through the generation of a single transcript that is post-transcriptionally processed to generate multiple functional products [13]. One example of a ncRNA which plays a role in canonical network is the highly abundant lncRNA metastasis-associated lung adenocarcinoma transcript 1 (MALAT1), which is overexpressed in lung, breast, and pancreatic cancers, etc. [21]. MALAT1 is retained in nuclear domains and associated with SC35 splicing domains, which are enriched in a large number of splicing factors and other factors involved in mRNA metabolism [22]. MALAT1 also functions as a precursor for the production of a small tRNA-like molecule termed MALAT1-associated small cytoplasmic RNA (mascRNA), which is localized in the cytoplasm [23]. MALAT1 is also expressed in normal thyroid (NT) and thyroid tumors [24], with increased expression during progression from NT to papillary thyroid carcinomas (PTC). However, MALAT1 expression was downregulated in poorly differentiated TC and ATC compared to NT [24]. Knockdown of MALAT1 inhibited cell proliferation and invasion of human thyroid tumor cell lines [25]. Upregulation of MALAT1 expression in PTC cells was induced by TGF-β mediated induction of epithelial-to-mesenchymal transition (EMT), suggesting a potential role of MALAT1 in EMT-mediated TC progression [24]. MALAT1 mediated FGF2 protein secretion from tumor-associated macrophages (TAMs); as a result it promoted the proliferation, migration, and invasion of TC cells and induced angiogenesis [26].

Some genetic loci produce numerous gene products from distinct transcripts and multiple gene products from the same transcript [13], suggesting that some ncRNAs may work as a coordinator of complex GRNs. H19 locus encodes the H19 lncRNA which can be processed to give rise to miR-675 [27]. miR-675 functions as a tumor suppressor in cancers [28,29]. The human H19 locus also encodes an antisense RNA [30]. H19 opposite tumor suppressor (HOTS) inhibits cell growth in a cervical cancer cell line, whereas its silencing promotes cell growth in vitro and tumorigenicity in vivo, indicating that it is an imprinted tumor suppressor [30]. Furthermore, the full-length H19 regulates the epigenetic state of some genetic loci [31]. H19 has both tumor-promoter and tumor-suppressive functions [32,33]. H19 is also expressed in TC samples and cell lines [34,35]. Overexpression of H19 in PTC cell lines increased cell proliferation, migration, and invasion, whereas its knockdown decreased cell viability and invasion in vitro and in vivo [34]. Moreover, knockdown of H19 inhibited tumor metastasis in vivo [35]. As a competitive endogenous RNA (ceRNA), H19 antagonized the function of miR-17-5p upon overexpression of its target *YES1* and suppressed miR-17-5p-induced cell cycle progression [34].

The lncRNA PTC susceptibility candidate 3 (PTCSC3) [36] plays a role as a ceRNA for miR-574-5p in TC cells (including ATC cells), and induces cell growth inhibition, cell cycle arrest, and apoptosis [37]. Additionally, PTCSC3 suppresses S100A4, VEGF, and MMP-9 expression in aggressive TC cells, leading to a reduction in cell invasion and motility [36].

## 5. Roles of ncRNAs in Non-Canonical Network Motifs

ncRNAs elicit networks in cancer cells through further motifs of interactions, which are called non-canonical network motifs [13]. ncRNAs can bind to proteins as well as protein complexes and regulate their function [38]. The direct binding of ncRNAs to proteins can promote their ability to target individual proteins or provide scaffolding for protein complexes to assemble [13], e.g. HOX transcript antisense RNA (HOTAIR) lncRNA [39]. HOTAIR promotes selective re-targeting of polycomb repressive complex 2 (PRC2) to an occupancy pattern and leads to genome-wide modification in the DNA histone H3 lysine 27 trimethylation of different genes that resemble epigenetic states found in early development (embryonic fibroblasts) [39]. Epigenetic modifications in cancer cells influence gene expression patterns and may support cancer invasion and metastasis [39]. HOTAIR expression is increased in TC and correlates with poor prognosis in TC [40]. Knockdown of HOTAIR significantly inhibits cell growth and invasion in TC cell lines [40]. Overexpression of HOTAIR promotes TC cell growth, migration, and invasion through inhibition of miR-1 and activation of cyclin D2 protein in PTC and follicular thyroid cancer (FTC) cell lines [41]. HOTAIR overexpression in PTC is linked to poor survival and may play a role in carcinogenesis via the Wnt signaling pathway [42]. Previous studies used a PTC-derived cell line (TPC1) and squamous carcinoma cell line (SW579) to elucidate the role of HOTAIR in aggressive TC [40]; knockdown of HOTAIR significantly inhibited cell growth and invasion [40].

Importantly, NEAT1 and MALAT1 bind and co-localize with hundreds of active genes in human cells, and transcriptional activity might influence NEAT1 localization [43]. Some studies reported that NEAT1 interacted with miR-129-5p or miR-214 in TC cell lines and influenced tumor cell survival, migration, and invasion [44,45].

Overall, the role of these ncRNAs and their transcriptional regulation in ATC or poorly differentiated thyroid cancer needs to be investigated further.

## 6. Future Directions

ncRNAs may be therapeutically targeted or agents of therapy. Also, they may be used as potential biomarkers for the diagnosis of thyroid cancer and the prediction of tumor aggressiveness (Figure 1). Further research will be needed to elucidate the GRNs (Figure 2) of ncRNAs (e.g., lincRNAs, miRNAs, etc.) in ATC.
